# Psychometric Properties and Cultural Adaptation of the Polish Version of the Healthy Lifestyle and Personal Control Questionnaire (HLPCQ)

**DOI:** 10.3390/ijerph18179190

**Published:** 2021-08-31

**Authors:** Michał Czapla, Raúl Juárez-Vela, Anna Rozensztrauch, Piotr Karniej, Izabella Uchmanowicz, Iván Santolalla-Arnedo, Alicja Baska

**Affiliations:** 1 Department of Public Health, Faculty of Health Sciences, Wroclaw Medical University, 51-618 Wroclaw, Poland; michal.czapla@umed.wroc.pl (M.C.); piotr.karniej@umed.wroc.pl (P.K.); 2 Institute of Heart Diseases, University Hospital, 50-556 Wroclaw, Poland; 3 Biomedical Research Center of La Rioja (CIBIR), Research Group in Care (GRUPAC) and Group of Research in Sustainability of the Health System, Department of Nursing, University of La Rioja, 26004 Logroño, La Rioja, Spain; ivan.santolalla@unirioja.es; 4 Department of Pediatrics, Division of Neonatology, Faculty of Health Science, Wroclaw Medical University, 51-618 Wroclaw, Poland; anna.rozensztrauch@umed.wroc.pl; 5 Department of Clinical Nursing, Faculty of Health Sciences, Wroclaw Medical University, 51-618 Wroclaw, Poland; izabella.uchmanowicz@umed.wroc.pl; 6 Center of Postgraduate Medical Education, Department of Lifestyle Medicine, School of Public Health, 01-813 Warsaw, Poland; alicja.baska@cmkp.edu.pl

**Keywords:** lifestyle, healthy lifestyle, health behaviour, life style

## Abstract

Background: Chronic non-communicable diseases (NCDs), sometimes referred to as lifestyle diseases, are the most common cause of death and disability worldwide. Thus, healthcare professionals should be equipped with tools, knowledge, skills, and competencies in the newly distinguished field of lifestyle medicine. The purpose of this study was to test the psychometric properties of the Polish version of the Healthy Lifestyle and Personal Control Questionnaire (HLPCQ). The Polish version of the HLPCQ would further provide Polish healthcare professionals with a useful and convenient tool for routine lifestyle assessment while giving HLPCQ novel use and potential for further research. Methods: Before testing its psychometric properties, the HLPCQ was translated and adapted from the original Greek version into Polish. Subsequently, we tested the instrument’s psychometric properties on a sample of 2433 participants. In addition, we tested the factorial validity of the HLPCQ using confirmatory and exploratory factor analysis. Results: There were more female than male participants (91.78%). Most of them were middle-aged (30.40 ± 7.71), single (39.62%), and living with family (70.65%). In terms of residence, 1122 (46.12%) participants lived in cities with a population of over 500,000. In terms of reliability, the internal consistency of the Polish version and its domains is excellent. Cronbach’s alpha for each of the domains of the scale ranged between 0.6 and 0.9. Conclusions: The Polish version of the Healthy Lifestyle and Personal Control Questionnaire (HLPCQ) has good characteristics of factorial validity and can be used in clinical practice and research.

## 1. Introduction

Chronic non-communicable diseases (NCDs), sometimes referred to as lifestyle diseases, are the most common cause of death and disability worldwide, accounting for nearly 70% of all deaths globally [1]. The modifiable behavioral risk factors underlying their occurrence are well studied. For example, the results of numerous studies show that over 90% of type 2 diabetes cases, over 80% of cardiovascular diseases, 50% of strokes, and over 30% of cancers could be prevented with a healthy lifestyle [2,3,4]. The importance of a healthy lifestyle was also in the spotlight in the face of the latest pressing challenge: the COVID-19 pandemic. In the United States, lifestyle-related diseases—obesity, type 2 diabetes, hypertension, and heart failure contributed to over 60% of COVID-19 hospitalizations [5]. Thus, lifestyle interventions, addressing usually the following six areas: nutrition, physical activity, exposure to risky substances (such as alcohol or tobacco), stress management, sleep habits, and social support & relationships should play a central role in modern healthcare.

The myriad of factors influencing personal lifestyle choices is still a subject of vivid discussion. For example, it is difficult to estimate precisely the contribution of conscious, autonomous decisions, socio-economic determinants or distinguish the impact of the obesogenic environment individuals inhabit [6,7]. Yet, there is a consensus that in the face of an oncoming “tsunami of common chronic diseases,” both systemic and individual-level interventions are needed [8,9].

To enable the latter, healthcare professionals in particular should be equipped with tools, knowledge, skills, and competencies in the newly distinguished field of lifestyle medicine [9,10].

At the same time, it is estimated that less than 40% of physicians routinely counsel their patients on lifestyle issues [11], while chronic diseases are the reason for 60–70% of all primary care visits in developed countries [9]. While it is beyond the scope of this study to describe the complex reasons for this “squandered opportunity”, as referred to by Rippe [11], it is worth mentioning a few of them: time demands, lack of reimbursement, and lack of knowledge and education in these areas among physicians.

The authors of this article recognize that routine assessments of patient lifestyles are necessary among the tools required to implement lifestyle interventions effectively. Such an evaluation should consider individual behaviors in crucial lifestyle areas (e.g., weekly level of physical activity) and measure health empowerment. It is well established in the literature that the internal health locus of control and a high level of self-efficacy are positively correlated with successful behavior change [12]. Indeed, psychological factors underpinning the behavior change process are mentioned in numerous guidelines on lifestyle diseases management [13], but few or no tools are provided to translate this knowledge into practice.

Considering the above, as well as the healthcare system’s limitations (for example, time constraints) ideally, a lifestyle assessment questionnaire should be short, feasible for routine use by physicians and other healthcare professionals, and comprehensive. According to the authors of this article, these criteria are met by the Healthy Lifestyle and Personal Control Questionnaire (HLPCQ) [12].

The goal of the questionnaire was “detecting and quantifying lifestyle patterns that reflect health empowerment and of the internal health locus of control,” which was proven to be achieved during psycometric tests using other available scales. Furthermore, given that no similar tool is available in Poland, this study aimed to perform transcultural adaptations and psychometric tests of the Polish adaptation of the HLPCQ. Thus, it provides Polish healthcare professionals with a useful and convenient tool for routine lifestyle assessment while giving the HLPCQ novel use and potential for further research.

## 2. Materials and Methods

### 2.1. HLPCQ

The Healthy Lifestyle and Personal Control Questionnaire (HLPCQ) is a 26-item tool. In it, the respondents are asked to indicate the frequency of adopting 26 positively stated lifestyle habits using a Likert-type scale (1 = never or rarely, 2 = sometimes, 3 = often, and 4 = always). There are 12 items concerning diet, 8 concerning daily time management, 2 referring to organized physical exercise, and 4 concerning practices of social support and positive thinking (e.g., positive thoughts during difficulties and emptying the mind during bedtime).

### 2.2. Translation, Adaptation, and Modeling 

Before testing its psychometric properties, the HLPCQ was translated and adapted from the original Greek version into Polish. We followed the guidelines of Beaton et al. [14]. The process included the following 6 steps (translation, synthesis, back translation, back translation synthesis, expert committee review of the translated version, and pretesting).

The original HLPCQ [12] was translated into Polish by 2 qualified translators. This Polish translation was blindly back-translated into Greek by bilingual translators who had not seen the original Greek version. Both translators were instructed to use simple sentences and avoid metaphors, colloquial terminology, passive sentences, and hypothetical statements. The authors of the original version of the HLPCQ did not submit any comments to the back-translated version. Subsequently, an expert committee compared and contrasted both the original and back-translated versions of the HLPCQ and agreed, by consensus, on a final Polish version of the HLPCQ. This expert committee consisted of nurses, dieticians, physicians (lifestyle medicine specialists), and public health specialists. 

As a final stage, cognitive interviews were completed on a sample of 40 volunteers. In this stage, some minor changes were introduced to the translation to improve the readability of the items. For example, to clarify the definition of soft drinks.

### 2.3. Population and Scope of the Study

A cross-sectional study was carried out on 2433 participants between February and March 2021. The participants completed an anonymous questionnaire distributed by social media (e.g., by Polish Society of Lifestyle Medicine). They were fully informed of the purposes of the study and expressed their consent. Inclusion criteria included reading and writing in Polish, at least 18 years old, and no physical or mental disability. The data were collected using a 2-section, self-administered questionnaire. The first section contained demographic data, such as age, gender, body mass index (BMI), place of residence, employment status, material status, educational level, etc. The second part was the Polish version of the Healthy Lifestyle and Personal Control Questionnaire (HLPCQ). The study was developed through the platform www.webankieta.pl to obtain layered data and access control of the sample using IP filtering.

### 2.4. Ethical Consideration

The study design and procedures were approved by the Bioethics Committee of Wroclaw Medical University in Poland (No. KB–207/2021).

### 2.5. Statistical Analysis 

The statistical analysis was performed using Stata 16 software (STATA Coorp.College Station, TX United States).

To assess the relevance of performing an exploratory factor analysis on the sample, the Kaiser-Meyer-Olkin (KMO) sample adequacy statistic and the Bartlett sphericity statistic were calculated beforehand. Analysis adequacy was determined by a KMO and Bartlett’s test of sphericity with a rejected null hypothesis of sphericity. EFA was performed according to determine the number of latent constructs and the underlying factor structure of the scale.

## 3. Results 

Table 1 presents the main characteristics of our sample. It included more women than men (91.78%). Most participants were middle-aged (30.40 ± 7.71), single (39.62%), and living with family (70.65%). In terms of place of residence, 1122 (46.12%) participants lived in cities with over 500,000 inhabitants. Approximately half of them were women, i.e., 1150 (47.27%), had a master´s degree and were employed, 129 (5.30%) unemployed, 17 (0.70%) retired, 462 (18.99%) students, and 1825 (75.01%) employed. 

In addition, 714 (29.35%) individuals were living alone, 458 (18.82%) had a shift job, most, i.e., 987 (40.57%), worked between 20 and 40 h per week, and 788 (32.39%) worked more than 40 h. 

The mean BMI was 23.69 Kg/m2 ± 4.59. In addition, 876 (36%) individuals reported a disease diagnosed by their physician, and the majority of patients, i.e., 1008 (41.43%), were considered as healthy as other people of the same age.The sample used for questionnaire testing was conducted with 20 patients per item of the questionnaire, to guarantee good psychometric values.

The results of the principal component analysis (PCA) of the 26 items with orthogonal rotation (varimax) are presented in Table 2. To assess the relevance of performing an exploratory factor analysis on the sample, the Kaiser-Meyer-Olkin (KMO) sample adequacy statistic and the Bartlett sphericity statistic were calculated beforehand. Analysis adequacy was determined with a KMO of 0.899 and Bartlett’s test of sphericity with a of <0.01

To ensure that the factorial model is adequate, an exploratory factor analysis (EFA) was performed using principal component analysis with a varimax rotation to determine the number of latent constructs and the underlying factorial structure of domains in the Polish version. The number of factors on the scale was estimated, considering two complementary criteria: 1) the Kaiser-Guttman or latent root criterion and 2) the drop contrast criterion.

In terms of reliability, the internal consistency of the Polish version and its domains is acceptable. For example, Cronbach’s alpha for each of the scale domains ranged between 0.6 and 0.9.

Table 3. presents the mean scores of each subscale along with the theoretical and observed values of the range. In addition, there was a good dispersion of calculated scores in our sample relative to the possible range of scores.

Table 4 presents the correlations between subscales. Specifically, all subscales were significantly positively correlated with each other, indicating that individuals adopting healthy dietary habits and avoiding dietary harms also follow a daily routine in their activities, exercise in an organized manner, seek social support, and care for their mental health.

## 4. Discussion 

The psychometric properties and cultural adaptation of the Polish version of the Healthy Lifestyle and Personal Control Questionnaire (HLPCQ) was carried out successfully. To our knowledge, this is the first study validating the HLCPQ in Polish and improving our understanding of the dimensions measured by the HLPCQ (daily routine, healthy dietary choices, social and mental balance, organized physical exercise, dietary harm avoidance), giving a culturally equivalent instrument to assess the efficacy of health-promoting interventions to improve individuals’ lifestyles and well-being. To date, similar psychometric test studies outside Greece were conducted only in Iran [15]. There were no language difficulties during the cross-cultural adaptation process, and only some expressions were modified slightly to ensure their cultural equivalence. The internal consistency of the Polish version of the complete scale and of each of the five domains that compose it is high, with values very close to those found in the original version of the HLPCQ. In our study, the five adopted domains explain almost 48.92% of the total scale variance, which is close to the original scale’s 46.69%.

It was proven that the HLPCQ shows acceptable reliability and is structurally accurate in Polish. Therefore, it can be applied as a useful and pertinent tool to assess individual behaviors corresponding to a healthy lifestyle and self-control. The study carried out in Iran included a group of 300 students of medicine, indicating the reliability of the alpha tool–0.78 [15]. In Poland, 2344 participants were included in the study, while in Greece–308.

The coefficient of the Polish version of the questionnaire for the five subscales selected during the analysis ranged from 0.606 to 0.852. Similar alpha values were reported by the authors of the original questionnaire, Darviri et al. [12]. For the daily routine subscale–Cronbach’s alpha was 0.852; healthy dietary choices–Cronbach’s alpha–0.661; social and mental balance–Cronbach’s alpha–0.777; organized physical exercise–Cronbach’s alpha–0.796; and dietary harm avoidance–Cronbach’s alpha–0.606. 

The number of health benefits that accrue due to healthy lifestyles continues to increase in number and importance. Many studies show that improvement in the above areas is essential to living in better conditions and improving quality of life [16,17,18,19,20,21]. Lifestyle interventions should also be a part of the response to COVID-19 and the prevention of future pandemics. Yet, an urgent need to implement this knowledge into practice remains poorly addressed [5,8,22]. Clinicians, public health practitioners, and other medical staff should be reassured that the benefits of lifestyle modification efforts are overwhelmingly positive and continue to grow, and we should renew our efforts to help patients add life to their years, as well as years to their life.

Taking that into account, the argument confirming the need for the adaptation of the HLPCQ was that a properly constructed tool for the synthetic analysis of lifestyle variables including self-control had not existed before in Poland. The overall psychometric performance of the HLPCQ is satisfactory. It can enhance outcome evaluations in future research, and could be recommended for epidemiological studies and primary care to measure the lifestyle patterns of individuals.

## 5. Conclusions

The results of this study of translation and adaptation into Polish of the Healthy Lifestyle and Personal Control Questionnaire (HLPCQ) suggest that the reliability and validity of the five dimensions are acceptable. Furthermore, the results are largely compatible with the initial hypothesis, what makes it useful for clinical and research use in our country. Compared with the rest of the psychometric tests, the results of transcultural adaptation were similar to the original Greek and Persian validation versions. As highlighted in the article, the effective implementation of lifestyle medicine should be a priority. It is worth pointing out that this would require multiple changes in current healthcare systems and public health policies. The HLPCQ could prove to be one of the useful tools in the process. 

## 6. Limitations

The study has some limitations that are important to highlight. The sample size, although sufficient to assess the main objectives of the study, could be improved by the addition of more participants. In addition, although we performed a descriptive observational study, carried out in a larger cohort of individuals to obtain the psychometric properties, we cannot demonstrate causality in the relationship between the variables studied.

The last limitation is that the study was conducted only via an online platform, possibly leading to the exclusion of some groups of potential participants (e.g., people without the internet or the elderly who usually use the internet rarely). 

## Figures and Tables

**Table 1:** Socio-demographic and health-related characteristics of the study sample (*N* = 2433).

Variable	*N* (%)	Mean	SD	Min.	Max.	P50
Age BMI	2433 2433	30.40 23.69	7.71 4.59	14 15.41	73 48.05	29.00 22.64
Gender Female Male	2233 (91.78) 200 (8.22)					
Place of residence City of 100,000–500,000 residents City of 20,000–100,000 residents City of more than 500,000 residents Town of up to 20,000 residents Rural area	431 (17.71) 347 (14.26) 1122 (46.12) 179 (7.36) 354 (14.55)					
Education level Bachelor’s degree PhD Master’s degree Elementary education Secondary education Vocational education	551 (22.65) 41 (1.69) 1150 (47.27) 39 (1.60) 639 (26.26) 13 (0.53)					
Employment status Full-time Business owner Part-time Retired Student Unemployed	1391 (57.17) 272 (11.18) 162 (6.66) 17 (0.70) 462 (18.99) 129 (5.30)					
Marital Status Divorced Informal relationship Married Single Widowed	63 (2.59) 614 (25.24) 785 (32.26) 964 (39.62) 7 (0.29)					
Who do you live with? Alone With family	714 (29.35) 1719 (70.65)					
In comparison with other people of the same age, how does the patient consider their health status? Better As good Does not know Not as good	498 (20.47) 1008 (41.43) 531 (21.82) 396 (16.28)					
Do you have a shift job? NO YES Unemployed	1555 (63.91) 458 (18.82) 420 (17.26)					
How many hours do you work per week? 20–40 h More than 40 h Up to 20 h Unemployed	987 (40.57) 788 (32.39) 169 (6.95) 489 (20.10)					
Do you have any chronic disease diagnosed by a physician? NO YES	1557 (64) 876 (36)					

SD: standard deviation, BMI: body mass index, p50: 50th percentile.

**Table 2:** Rotated factor loading of the principal component analysis (PCA) for 26 health-related lifestyle habits (N = 2433).

Items “How often…”	Healthy Dietary Choices	Dietary Harm Avoidance	Daily Routine	Organized Physical Exercise	Social and Mental Balance
“are you careful about how much food you put on your plate?”	0.2625				
“do you check the food labels before buying a product?”	0.3860				
“do you calculate the calories in your meals?”	0.4220				
“do you limit fat in your meals?”	0.5015				
“do you cook?”	0.2478				
“do you eat organic foods?”	0.3227				
“do you eat whole-wheat products?”	0.2273				
“do you avoid eating packaged- or fast food?”		0.3594			
“do you avoid soft drinks?”		0.4260			
“do you avoid eating when stressed or disappointed?”		0.5955			
“do you avoid binge eating when you are out with friends?”		0.6491			
“do you eat your meals at the same time each day?”			0.3296		
“are you careful about not missing a meal each day?”			0.2969		
“do you eat a good breakfast?”			0.2482		
“do you sleep at the same time each day?”			0.4060		
“do you follow a scheduled program for your daily activities?”			0.3357		
“do you eat breakfast at the same time each day?”			0.4317		
“do you eat lunch at the same time each day?”			0.3917		
“do you eat dinner at the same time each day?”			0.3889		
“do you do aerobic exercises for 20 or more minutes at least three times a week?”				0.4960	
“do you exercise in an organized manner?”				0.4310	
“do you share your problems or worries with others?”					0.2482
“do you concentrate on positive thoughts during difficulties?”					0.5940
“do you empty your head of thoughts or the next day’s program during bedtime?”					0.3895
“do you care about meeting and discussing with your family on a daily basis?”					0.4628
“do you balance your time between work, personal life, and leisure?”					0.4191
Eigenvalues	1.981	1.178	6.567	1.273	1.872
% of variance	7.62	3.94	25.26%	4.90	7.20
Cronbach´s alpha	0.661	0.606	0.852	0.796	0.777

Note: The translation of the items from the Polish language is presented only for interpretation and NOT for use in studies or clinical practice. For clinical practice, use the Polish version of the Healthy Lifestyle and Personal Control Questionnaire (Table S1) in Supplementary Materials.

**Table 3:** Descriptive characteristics of the five subscales and the total score of HLPCQ.

	Items	Range	Mean	SD	Min.	Max.
Daily routine	8	8–32	19.82	4.77	8	32
Healthy dietary choices	7	7–28	14.57	2.77	6	23
Social and mental balance	5	5–20	11.87	2.77	5	20
Organized physical exercise	2	2–8	4.29	1.73	2	8
Dietary harm avoidance	4	4–16	10.39	2.16	4	16
Total Score	26	26–104	60.96	10.22	31	93

HLPCQ: Healthy Lifestyle and Personal Control Questionnaire, SD: standard deviation.

**Table 4:** Correlations (Pearson´s rho) between HLPCQ subscales.

	Daily Routine	Healthy Dietary Choices	Social and Mental Balance	Organized Physical Exercise	Dietary Harm Avoidance
Daily routine	1				
Healthy dietary choices	0.4906 *	1			
Social and mental balance	0.3523 *	0.2262 *	1		
Organized physical exercise	0.4243 *	0.4178 *	0.2910 *	1	
Dietary harm avoidance	0.3513 *	0.4390 *	0.1916 *	0.2983 *	1

* Correlation is significant at the 0.05 level (2-tailed). HLPCQ: Healthy Lifestyle and Personal Control Questionnaire.

## Data Availability

The data will be available upon contacting the corresponding author.

## Supplementary Materials

The following are available online at www.mdpi.com/1660-4601/18/17/9190/s1, Table S1: The Polish Version of Healthy Lifestyle and Personal Control Questionnaire.
