# Supplementary material for: Psychometric Properties and Cultural Adaptation of the Polish Version of the Healthy Lifestyle and Personal Control Questionnaire (HLPCQ)
Source: Int J Environ Res Public Health. 2021 Aug 31;18(17):9190. doi: 10.3390/ijerph18179190 (PMC8431541; doi:10.3390/ijerph18179190)
Supplement: Supplementary file 1 [file ijerph-18-09190-s001.zip › ijerph-1341685-SI.pdf]

Supplementary Material
Table S1. The Polish version of the Healthy Lifestyle and Personal Control Questionnaire

Kwestionariusz Zdrowego Stylu Życia i Samokontroli

Poniżej znajdziesz zestaw codziennych zachowań i nawyków.

Wskaż, jak często obserwujesz u siebie te zachowania na podstawie następującej skali:

1 = rzadko lub nigdy, 2 = czasami, 3 = często, 4 = zawsze

1. Zwracam uwagę na ilość jedzenia na moim talerzu	1	2	3	4
2. Staram się jeść codziennie o tej samej porze	1	2	3	4
3. Sprawdzam etykiety produktów, które kupuję	1	2	3	4
4. Liczę kalorie w posiłkach	1	2	3	4
5. Ograniczam tłuszcze w posiłkach	1	2	3	4
6. Dbam, aby nie opuścić żadnego posiłku w ciągu dnia	1	2	3	4
7. Dbam o to, żeby jeść dobre śniadanie	1	2	3	4
8. Unikam gotowych posiłków / posiłków typu '*fast food*'	1	2	3	4
9. Unikam słodzonych napojów	1	2	3	4
10. Kiedy jestem zestresowany/-a lub zdenerwowany/-a, unikam jedzenia dla ulgi	1	2	3	4
11. Unikam jedzenia w dużej ilości, kiedy jestem z przyjaciółmi lub kiedy wychodzę	1	2	3	4
12. Chodzę spać codziennie o tej samej porze	1	2	3	4
13. Lubię gotować	1	2	3	4
14. Jem produkty pochodzenia organicznego (ekologiczne)	1	2	3	4
15. Jem śniadanie codziennie o tej samej porze	1	2	3	4
16. Jem produkty pełnoziarniste	1	2	3	4
17. Jem obiad codziennie o tej samej porze	1	2	3	4
18. Dzielę się swoimi problemami i troskami z innymi	1	2	3	4
19. Jem kolację codziennie o tej samej porze	1	2	3	4
20. Wykonuję ćwiczenia aerobowe (np. chodzenie, jazda na rowerze) przez 20 minut lub więcej co najmniej 3 razy w tygodniu				
21. W trudnych chwilach dbam o to, żeby skupiać się na pozytywnych myślach	1	2	3	4
22. Moje codzienne aktywności przebiegają według określonego harmonogramu	1	2	3	4
23. Mam ustalony program ćwiczeń/treningów, zgodnie z którym ćwiczę	1	2	3	4
24. Kiedy zasypiam oczyszczam umysł z myśli i nie zastanawiam się nad planem na jutrzejszy dzień.	1	2	3	4
25. Dbam o częste spotkania i rozmowy z osobami z mojego środowiska rodzinnego	1	2	3	4
26. Zachowuję równowagę między pracą, czasem wolnym i życiem osobistym	1	2	3	4
**Instrukcja dotyczące punktacji**<br><br>**Podskale**<br><br>**A. Zdrowe wybory żywieniowe (Healthy dietary choices)**<br><br>1+3+4+5+13+14+16				

**B. Dietetyczne unikanie szkód (Dietary harm avoidance)**<br>8+9+10+11<br>**C. Codzienna rutyna (Daily routine)**<br>2+6+7+12+15+17+19+22<br>**D. Zorganizowane ćwiczenia fizyczne (Organized physical exercise)**<br>20+23<br>**E. Wsparcie społeczne i zdrowie psychiczne (Social and mental balance)**<br>18+21+24+25+26<br>**Ogólna ocena= A+B+C+D+E**				
Czapla, M.; Juárez-Vela, R.; Rozensztrauch, A.; Karniej, P.; Uchmanowicz, I.; Santolalla-Arnedo, I.; Baska, A. Psychometric Properties and Cultural Adaptation of the Polish Version of the Healthy Lifestyle and Personal Control Questionnaire (HLPCQ). *Int. J. Environ. Res. Public Health* **2021**, *18*, 9190. https://doi.org/10.3390/ijerph18179190				
